# Competing priorities: a qualitative study of how women make and enact decisions about weight gain in pregnancy

**DOI:** 10.1186/s12884-020-03210-5

**Published:** 2020-09-03

**Authors:** Meredith Vanstone, Marina Sadik, Sherry Van Blyderveen, Anne Biringer, Wendy Sword, Louis Schmidt, Sarah D. Mcdonald

**Affiliations:** 1 Department of Family Medicine, McMaster University, 1280 Main Street W, Hamilton, Ontario L8S 4L8 Canada; 2 Department of Family Medicine, DBHSC 5003E, McMaster University, 100 Main St. W, Hamilton, ON L8P 1H6 Canada; 3 Eating Disorders Program at Homewood Health Centre, 150 Delhi Street, Guelph, Ontario N1E 6K9 Canada; 4 Ray D. Wolfe Department of Family Medicine, Mount Sinai Hospital, 600 University Ave, Toronto, Ontario M5G 1X5 Canada; 5 School of Nursing, McMaster University, 1280 Main Street W, Hamilton, Ontario L8S 4L8 Canada; 6 Department of Psychology, Neuroscience & Behaviour, McMaster University, 1280 Main Street W, Hamilton, Ontario L8S 4L8 Canada; 7 Division of Maternal-Fetal Medicine, Department of Obstetrics and Gynecology, McMaster University, 1280 Main Street W, Hamilton, Ontario L8S 4L8 Canada; 8 Department of Health Research Methods, Evidence & Impact, McMaster University, 1280 Main Street W, Hamilton, Ontario L8S 4L8 Canada; 9 Department of Radiology, McMaster University, 1280 Main Street W, Hamilton, Ontario L8S 4L8 Canada

**Keywords:** Pregnancy, Gestational weight gain, Qualitative methods, Prenatal care, Counselling

## Abstract

**Background:**

Despite ample clinical evidence that gaining excess weight in pregnancy results in negative health outcomes for women and infants, more than half of women in Western industrialized nations gain in excess of national guidelines. The influence of socio-demographic factors and weight gain is well-established but not causal; the influence of psychological factors may explain some of this variation.

**Methods:**

This is the qualitative portion of an explanatory sequential mixed-methods study designed to identify predictive psychological factors of excess gestational weight gain (QUAN) and then explain the relevance of those factors (qual). For this portion of the study, we used a qualitative descriptive approach to elicit 39 pregnant women’s perspectives of gestational weight gain, specifically inquiring about factors determined as relevant to excess gestational weight gain by our previous predictive study. Women were interviewed in the latter half of their third trimester. Data were analyzed using a combination of unconstrained deductive content analysis to describe the findings relevant to the predictive factors and a staged inductive content analytic approach to examine the data without a focus on the predictive factors.

**Results:**

Very few participants consistently made deliberate choices relevant to weight gain; most behaviour relevant to weight gain happened with in-the-moment decisions. These in-the-moment decisions were influenced by priorities, hunger, a consideration of the consequence of the decision, and accommodation of pregnancy-related discomfort. They were informed by the foundational information a woman had available to her, including previous experience and interactions with health care providers. The foundational information women used to make these decisions was often incomplete. While women were aware of the guidelines related to gestational weight gain, they consistently mis-applied them due to incorrect understanding of their own BMI. Only one woman was aware that weight gain was linked to maternal and infant health outcomes.

**Conclusions:**

There is an important role for prenatal providers to provide the foundational information to positively influence in-the-moment decisions. Understanding how weight gain guidelines apply to one’s own pre-pregnancy BMI and comprehending the well-established link between gestational weight gain and health outcomes may help women prioritize healthy weight gain amongst many competing factors.

## Introduction

There is ample clinical evidence that gaining excess weight during pregnancy results in negative health outcomes for women and infants. Women who gain excess gestational weight face increased risk of caesarean delivery and associated complications [1] and are less likely to return to their pre-pregnancy weight [2–5]. They are more likely to experience hypertension and diabetes both during and after pregnancy [6–9]. Infants whose mothers gained in excess of guidelines have a risk of metabolic disorders and higher weight later in life [10–14]. They are more likely to be large for gestational age, which carries concomitant risk of pre-term delivery, trauma at birth, and neonatal intensive care unit admission [8, 15–18].

Despite the well-known nature of these risks, more than half of women in Western countries gain in excess of gestational weight gain guidelines [1, 19–23]. The rate of women who gain in excess of clinical guidelines is increasing with time [24, 25]. The relevance of socio-demographic and structural factors to excess weight gain is well established. Outside of pregnancy, obesity is correlated with factors such as low income and education levels, [26, 27] living in a socioeconomically disadvantaged neighborhood, [26, 28–30] experiencing chronic stress and depression, [31, 32] and perceiving low levels of social support [33, 34]. These findings concord with evidence about weight gain in pregnancy and pregnant women’s perceptions of barriers to and facilitators of appropriate gestational weight gain [35–45].

However, not all pregnant women who experience these barriers gain weight in excess of guidelines, and many women who do not experience these barriers do gain in excess. This suggests the importance of psychological and behavioral factors. In a recent prospective cohort study, we found that the following individually-relevant features are predictive of excess gestational weight gain: nulliparity, being overweight, planning excessive gain, eating in front of a screen, low self-efficacy about pregnancy weight gain, thinking family and friends believe pregnant women should eat twice as much as before pregnancy, being agreeable, and having emotion control difficulties [46]. While this study determined the predictive relevance of these factors, it could not explain *why* these factors were relevant, or how they related to each other.

We therefore set out to conduct a qualitative study of women’s weight-related thoughts and behaviors, with the objective of understanding how women make and enact decisions about weight gain during pregnancy. We conducted interviews with women in their third trimester, asking them about their weight-gain related practices and habits during pregnancy. As planned at the outset of the original study, we queried participants specifically about the factors shown to be predictive or protective of excess gestational weight gain (GWG) in our previous study [46].

## Methods

This is the qualitative portion of an explanatory sequential mixed-methods study [47] designed to identify psychological factors predictive of excess gestational weight gain (QUAN) [46] and then explain the relevance of those factors (qual). For this portion of the study, we used a qualitative descriptive approach [48–50] to elicit pregnant women’s experiences, thoughts, and opinions on gestational weight gain, specifically inquiring about factors determined as relevant to excess gestational weight gain by our previous cohort study [46]. The pairing of qualitative and quantitative methods is useful because while the quantitative study identifies which aspects are predictive of excess weight gain, it is not able to offer explanation of *why* or *how* these factors matter. The exploratory aspect of the qualitative work can query these factors in greater detail to help understand how women may experience, navigate, or respond to these predictive factors.

### Research team

This study was conducted by a multi-disciplinary research team with complementary expertise in psychology (SVB, MS, LS), obstetrics (SM), family medicine (AB), and qualitative research in the area of perinatal health (MS, MV, WS). We also have previous experience conducting research about weight gain and/or with pregnant women. We approached this research from a pragmatic philosophical perspective and therefore sought the meaning of ideas and values by looking at their outcomes and practices in which they are embedded [51]. This approach strikes a balance between appreciating that weight and notions of “appropriate” weight gain are socially constructed concepts while also recognizing the significant scientific evidence that exists about the mechanisms of weight gain and the health-related outcomes correlated with excess gestational weight gain.

### Sample and recruitment

We recruited women in the latter half of their third trimester for individual interviews. We excluded women who had already given birth, to ensure strong recall of information on behaviours, lifestyles, and decision-making processes. Participants were eligible to participate if they were in the third trimester of a viable singleton pregnancy, were currently living in Ontario, and could participate in an interview conducted in English.

Pregnant women were recruited in a variety of ways. We used social media to post advertisements in pregnancy and parenting forums. We also recruited through prenatal clinics run by obstetricians (OB), family physicians (FP) and midwives (MW). Each clinic chose to operationalize recruitment differently, either by posting our advertisement in their exam rooms, by handing out “consent to contact” forms, or a combination of the two. When contacted by a potential participant, we asked a short number of screening questions to establish eligibility. We began with a convenience sample, accepting all interested participants who met the eligibility criteria. These screening questions were designed to ensure that participants would have the necessary experience to yield rich data, and also allowed us to monitor the composition of our initial sample so that we could later purposively sample for particular characteristics (e.g. range of pre-pregnancy body weights) if that variation did not occur in our initial convenience sample. After deductive and inductive analysis of initial interviews, we employed purposive sampling to recruit participants who were able to speak to emerging ideas of analytic interest, using our screening questions, snowball sampling and personal networks to recruit participants with features of theoretical interest (e.g. pre-pregnancy BMI, age, education level). Data was collected past the point of saturation, to allow all participants who indicated interest the opportunity to share their thoughts and opinions. We defined saturation as the amount of data needed until nothing new was apparent and informational redundancy was reached [52, 53]. We required a relatively large number of interviews to reach this, given the different features we were seeking to include in our sample (e.g. variation in type of health care provider, pre-pregnancy body weight, weight gain trajectory, education level, parity). Three additional interviews conducted after we identified saturation, which allowed us to test that saturation had in fact been achieved. Participants received a $20 gift certificate to partially compensate for their time; any costs of travel or parking incurred for in-person interviews were covered.

### Data collection

We conducted individual interviews with women in their third trimester of pregnancy. Interviews were conducted by MS, a non-clinician qualitative researcher. Interviews were conducted in the latter half of the third trimester, when women would have gained most of their gestational weight.

We chose to conduct individual interviews in recognition of the sensitivity of the research topic, and the individual nature of a woman’s relationship to her body, pregnancy, and weight gain. A semi-structured interview guide was used, developed in collaboration with the predictive cohort study research team, and piloted with one pregnant woman. The interview guide (Additional file 1) included general queries about weight-related choices, questions about how the participant had handled particular scenarios, and questions specifically about the relevance of the factors identified as predictive of excess weight gain. The interview guide was refined over the course of the study, allowing us to more deeply explore areas of analytic relevance in earlier data collection. We offered participants the choice of conducting the interview in-person, by phone, or by video-conference. Interviews lasted 16–42 min (28 min on average), were recorded with permission, and transcribed verbatim.

During the interview, we asked each participant to provide her height, pre-pregnancy weight, current gestational age, and current weight. If women did not know this information, we asked them to re-contact us after their next medical visit. All numbers were self-reported; height and weight information were obtained from all participants.

### Data analysis

We used participant-provided height, pre-pregnancy weight, current gestational age, and current weight to calculate each person’s pre-pregnancy BMI (BMI = kg/m^2^) and current weight gain. We used rate of weight gain ([weight gain/gestational age] – 13 weeks) to calculate a prediction for total weight gain in pregnancy based on current weight gain and gestational age at the time of the interview. The pre-pregnancy BMI and prediction of total weight gain in pregnancy allowed us to categorize each participant as gaining within, above, or below gestational weight gain guidelines, according to the range established by the Institutes of Medicine [54]. This calculation was used to understand the composition of our sample, for readers to judge transferability, to explore findings related to pre-pregnancy weight as a predictive factor, and contextualize participant remarks within their personal trajectory of weight gain.

We conducted two types of qualitative data analysis. To examine the relevance of factors identified as predictive of excess gestational weight gain in our previous study, we employed an unconstrained deductive content analysis, [55] looking for data relevant to these particular features and grouping them for later inductive analysis to identify explanatory factors present in the data. Within these categories, and in the dataset not identified as relevant to the predictive factors we used an inductive approach to analysis, following an adapted form of the staged-coding technique of Grounded Theory [56]. We adapted the technique to suit the current methodology by remaining in a descriptive mode of analysis. Initial analysis began with open, line-by-line coding. This preliminary round of coding served to categorize each participant by features such as parity, education level, pre-pregnancy BMI and predicted weight gain. This allowed us to compare findings across participants grouped by type of health care provider, parity, pre-pregnancy BMI, or predicted weight gain. These groups were identified from the previous prospective study, and inductively as analysis proceeded. Charmaz acknowledges the likelihood that analysts will have relevant knowledge of evidence and theories that influence what they identify and prioritize in inductive analysis; our familiarity with the previous predictive study likely influenced the way we understood and interpreted the data during the inductive phase [56]. The two main analysts (MS and MV) remained open to contradictory findings by engaging in critical dialogue with each other at each point of analysis. We are confident in the credibility of our inductive findings because we did identify some discrepant and contradictory findings, particularly around the factors of parity and eating in front of a screen.

We engaged in triangulation of data, comparing findings across participants, particularly within and across groups of participants who had identified differences (e.g. weight gain trajectory, parity, pre-pregnancy body size) [57].

## Results

Thirty-nine women participated in this study in the third trimester of pregnancy. Their demographic features are described in Table 1, with weight-related information available in Table 2.

**Table 1:** Demographic information

	# (%) (*n* = 39)
**Education Level**	
High School or less	0 (0%)
University	18 (46%)
College or equivalent	9 (23%)
Post-graduate or Professional	11 (28%)
**Race**	
White	28 (72%)
Asian	4 (11%)
Mixed-race	2 (5%)
Indigenous	1 (3%)
Caribbean	1 (3%)
Middle Eastern	1 (3%)
**Prenatal Health Care Provider**	
Obstetrician (OB)	16 (41%)
Midwife	15 (39%)
Shared Care (FP + OB)	8 (21%)
Family Physician (FP)	0 (0%)
**Parity**	
Nulliparous	18 (46%)
Multiparous (1 previous birth)	15 (39%)
Multiparous (2/+ previous birth)	6 (15%)
**Age**	
< 24	1 (2%)
25–29	3 (7%)
30–34	18 (46%)
35–39	15 (38%)
> 40	2 (5%)

**Table 2:** Weight Profiles of Participants

	Percentage (n = 39)
**Predicted total pregnancy weight gain from weight at time of interview.**	
Gaining above guidelines	24 (62%)
Gaining within guidelines	7 (18%)
Gaining below guidelines	6 (15%)
**Pre-pregnancy BMI**	
Obese (>30)	7 (18%)
Overweight (25–29.9)	6 (15%)
Normal (18.5–24.9)	26 (67%)
Underweight (< 18.5)	0 (0%)

Table 1 reports participant demographic information.

Table 2 reports pre-pregnancy BMI distribution of participant, and uses antepartum weight at the time of the interview to predict whether total weight gain in pregnancy would be within, above, or below IOM guidelines.

### Findings

**Fig. 1:**
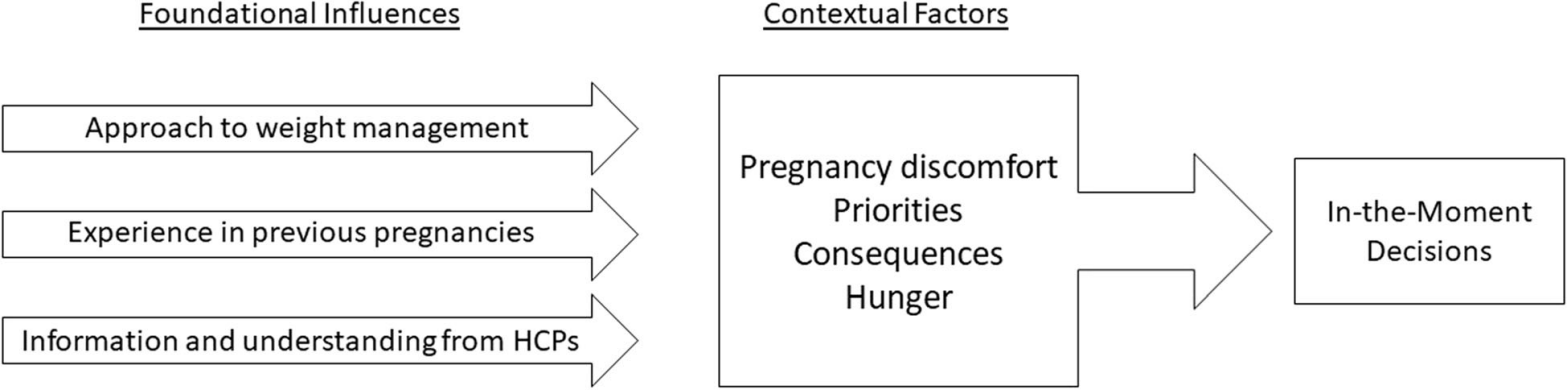
Decisions relevant to weight-gain in pregnancy are made many times each day. These decisions include choices about what to eat, when, and in what portion. These decisions are often made or re-made in-the-moment, and are informed by both foundational influences and contextual factors. The contextual factors depicted here are commonly relevant, but may not all be at play in each individual decision

Most participants did not perceive that they consciously formed and enacted decisions about weight gain during pregnancy; they were unable to speak at length about deliberate weight-related behaviours or choices. Instead, women reported other priorities during this time such as caring for existing children, alleviating physical discomfort, preventing miscarriage, and improving the quality of food they consumed. Prompted by semi-structured interview questions, participants described how their behaviours and choices were informed by foundational influences and varied according to in-the-moment circumstances (Fig. 1). The in-the-moment decisions were typically responsive to circumstance, rather than planned or proactive. A minority of participants took more active or deliberate approaches to weight management, also informed by foundational influences and in response to current circumstances. Few women reported receiving information about their level of weight gain as their pregnancy progressed. Those who became aware they were gaining in excess of guidelines found this information de-motivating with regards to weight management activity.

### Foundational influences of weight-related decisions

Each woman described experiences during and prior to pregnancy which influenced her understanding of the importance of weight control and informed her approach to weight during pregnancy. These foundational influences included pre-pregnancy approaches to weight management, experiences from previous pregnancies, and interactions with health care providers.

#### General approach to weight management

Women described their general approaches to weight management during and prior to pregnancy. These approaches were grouped in three categories: relaxed approaches to healthy weight gain, unconcerned approaches which did not consider weight management, and active attempts to manage weight gain. Most participants clustered in the relaxed and unconcerned categories; participant’s pre-pregnancy weight and weight gain trajectory did not seem to be relevant to their approach to weight management. We observed that most women did not change their pre-pregnancy approach to weight management after becoming pregnant; some became less concerned with weight management. We did not have any participants who became *more* concerned with weight management during pregnancy; the few who exhibited active approaches to manage weight gain during pregnancy described this as congruent with their pre-pregnancy approach to weight.

The relaxed approach to weight management was marked by women who talked generally about attempts to live a healthy lifestyle but were unable to give specific examples of what they do to stay healthy aside from “*try to eat healthy*” (P37) and “*don’t eat junk food*” (P38). Relaxed approaches were aware of and identified a desire to manage weight, but descriptions of action were vague and general without specific detail of changes made or actions taken. A typical relaxed attitude to weight management was described by one woman:

*“I eat healthy as much as I can, I mean I do have the occasional day where I don’t eat healthy. But I mean, most of the time I eat a pretty healthy balanced diet, take my vitamins, that’s really all that I can do.” (P16)*

Women who exhibited an unconcerned approach often had not thought about weight gain prior to the interview for this study and did not identify weight management as a goal. These women typically had not attempted to control their weight prior to pregnancy, and it did not occur to them that they should consider this issue during pregnancy. *“It’s not really something I focus on in a lot of ways, especially when pregnant, because if I think about it too much, I feel like I might get upset” (P16).*

Women who exhibited an active attempt at managing their weight often struggled with weight before pregnancy or in a prior pregnancy. Attention to nutrition and weight was typically prompted by some additional circumstance, such as diabetes or body image. Women who took an active approach to weight management were accustomed to thinking and planning their food and physical activity before pregnancy, and continued these behaviours during pregnancy.

*“So initially, I tried to portion down compared to what I would have portioned previously because I had less activity and I very clearly knew that. So, for example, if a target was 15 carbs for some fruits or something at breakfast, I would go with a plan of trying to hit 12 carbs, it’s not like a huge drop but enough that I would up my protein, I would have a high protein Greek yogurt, and a little bit less of the berries or I would choose different berries, let’s say blackberries and raspberries rather than strawberries or blueberries.” (P24)*

#### Experiences in previous pregnancies

Multiparous women described two main approaches to weight management. The first group included women who had taken a more vigilant approach in previous pregnancies. They relaxed their behavior in subsequent pregnancies, attributing the pressure of having other children and previous positive outcomes as a reason not to maintain the same vigilance:

*“Definitely I think more so the first pregnancy because I had a lot more time to be concerned about that kind of thing* [identifying a weight goal], *and I think you just you think a lot about everything in your first pregnancy right? So I think my first pregnancy I did a lot more research around what I should be eating, I was much more concerned about what was being consumed in that time. And I did have a lot more time and ability to control that whereas now I have two kids that are two and three, so I’m very busy.” (P5)*

Another group of multiparous women became more vigilant in later pregnancies. In their first pregnancy, these women were less stringent because they “[weren’t] *sure how their body would react to the pregnancy*” (P6). They felt more open minded the first time, trying to get a “feel” for how the pregnancy would change their body. In their second pregnancies, they were more likely to create goals and regimens related to weight gain, typically responding to previous experiences:

*“In my previous pregnancy I gained almost 60 pounds. And with this pregnancy, so far, I’ve gained about 40. It is definitely something that weighs on my mind a little bit, from time to time … I vowed not to gain as much weight this time around, and I wanted to gain kind of what the expected amount was” (P14).*

Participants who were caring for children described feeling limited in the types of food they cooked and ate. They also reported being more tired from caring for other children and connected this to a willingness to eat easy and accessible foods. Time and exhaustion were both factors that made food planning, preparation, and exercise more challenging for women. *“This one is a little bit different. I still try to eat healthy but it’s certainly not working out nearly as much as the first time back at work and taking care of him and all of that.”* (P21).

#### Information from health care providers

Almost all participants reported only cursory conversations about weight with their current prenatal health care provider (HCP), typically limited to the HCP notifying them of the IOM weight gain guideline. This finding was consistent across all types of health care provider. As a result, all but two participants were aware of the weight gain guidelines. Awareness of the guidelines did not mean correct application. Some women quoted guidelines for ‘normal’ BMI when the information they provided about their pre-pregnancy weight and height would categorize them as ‘overweight’ or ‘obese’. For example, after gaining 30 lbs. at 35 weeks, one woman characterized herself as in “*the good range*” (P16), between 25 and 35 lbs. However, her pre-pregnancy weight and height described an overweight BMI, which would mean her recommended weight gain range at the end of pregnancy was 15–25 lbs.

All but one participant reported that discussions about weight gain guidelines with their HCP did not include risks and benefits of gaining above or below the guidelines. Only one participant was aware of the risks involved with gaining over the recommended amount; her awareness was the result of a traumatic labour and delivery in a previous pregnancy: *“Something that I was told after my last pregnancy kind of sat with me. And that is: the more weight you gain, the more difficult your labor and your delivery can be. And you could end up having more damage and difficulties.” (P14).*

### Enacting weight-related decisions in-the-moment

The second aspect of weight management refers to the many daily decisions that affect weight gain. The foundational influences of weight gain set the stage for how these in-the-moment decisions are made, but when describing the pattern of decision-making women identified four strong influences: priorities, hunger, a consideration of the consequence of the decision, and accommodating pregnancy-related discomfort.

#### Priorities

Women’s in-the-moment decisions are influenced by their overall priorities during pregnancy, as well as their priorities in the moment of decision-making. Named priorities included general phrases about healthy pregnancy and healthy babies, caring for other children, reducing stress, managing other health conditions, and preventing miscarriage. Comments about healthy pregnancies and health babies were not linked to weight management. Some women offered explicitly that weight control was not a priority; not something they were motivated or able to control:

*“I closely watched my weight prior to pregnancy, and when you’re pregnant, you try to, and then you get to the point where you just throw it all out of the window, because you feel like you don’t really have that much of control over it. And you don’t have the same motivation, right, because you are like, “Ok well, putting on weight is what you are supposed to be doing” to a certain extent” (P05)*

#### Hunger

Women made it clear that they felt hunger was a signal that they were required to eat, with some women remarking that the baby was at risk if they did not eat when hungry: *“I need to listen to my body and when it needs something, I need to provide it.” (P25)* Many participants noted that they became cranky or upset when they felt hungry for long periods of time, so they learned to avoid the mood disruption by being prepared to eat at any time: *“I carry stuff with me, all the time. I’m in court a lot, for my job, so I’ve got tons of snacks all in my bag and stuff, because if I don’t, then I start to feel sick and stupid.” (P4).*

#### Considering consequences

We asked participants to describe their thought processes when faced with a weight-related decision such as eating unhealthy but delicious food. Women often described choosing to eat the dessert, but mentally deferring the consequence of that choice to after pregnancy. *“the more you put on before you have the baby, the more you have to take off after … it’s temporary, I’ll fix it later, I’ll do it later, I’ll clean up later” (P5)* This attitude was consistent in the majority of participants, who expressed that the main consequence of weight gain during pregnancy was the need to lose it afterwards: *“I’ll worry about it after when I have to lose the weight.” (P14)* Only one woman identified an understanding that excess weight gain was related to maternal and infant health outcomes, this knowledge came after a traumatic labor in a previous pregnancy.

#### Pregnancy discomfort

The discomforts associated with pregnancy were consistently reported as a challenge to decisions about food intake and physical activity. Women described nausea, vomiting, bloating, being a larger size, and swollen body parts as influencing their food and activity decisions: *“Unfortunately, because of physical change and things like that, I wasn’t able to be physically active anymore, so I ended up gaining weight very quickly.” (P20)* Women who experienced significant nausea and vomiting reported eating anything they could tolerate, without concern for nutritional or caloric value. *“I felt nauseous a lot, and the only thing that I felt I could eat, or wanted to eat, was carbohydrates and sugars.” (P14)* Participants also linked indulgence in food as a comfort and pleasure during a time which can be stressful, and where many typical indulgences (e.g. alcohol) are no longer available: *“Being pregnant is a pretty traumatic experience to your body, psychologically, and I think there’s only certain things that you can find pleasure in for this really kind of traumatic experience.” (P20).*

This sentiment of psychological or physical discomfort overriding rational decision-making was common, even among women with significant knowledge about health and nutrition. One participant with postgraduate education in nutrition summarized her experience eloquently: *“The psychological factors of being pregnant overpower the will power and education I have in nutrition” (P23).*

### Responding to information about success of the plan

Some women received feedback about their weight gain during pregnancy, typically when their HCP made a comment after recording their weight during a medical appointment. The ways in which women responded to the information about weight gain differed.

#### Response to excessive weight gain

Few women in our study received feedback about weight gain in their current pregnancy. While many women reported being weighed regularly by health care providers, most reported that their HCP was not concerned so they themselves were not concerned either, assuming that if there was a problem the HCP would raise it. *“They just write it down and they don’t say anything about it – so when they don’t say anything, I just assume it’s normal.” (P13)* This was an important theme, with many women remarking that they relied upon their HCP to identify weight as a problem, but seldom received information about this issue.

The small number who knew they were gaining too much weight were reluctant to try and alter behavior to decrease weight gain or lose weight during pregnancy. None of the women who were alerted to excess weight gain during pregnancy received instructions or advice on how to manage this. They typically described weight management in terms of “losing weight”, believing that this would be unhealthy for the baby: *“I am too nervous right now to try to do any, like, mitigation or cut calories or anything like that, because I just don’t have enough information and/or wouldn’t think it’s ideal to start restricting at this point.” (P23)* Others found the weight gain to be de-motivating, and ceased monitoring their weight and food intake because they felt discouraged and out of control: “*I ended up gaining weight very quickly and like a pretty significant amount of it as well, and then I stopped checking my weight.” (P20).*

## Discussion

Our study shows that few pregnant women prioritize healthy weight gain in pregnancy; most are concentrating on other goals and objectives. When asking women how they made and enacted decisions that would influence weight gain, it became apparent that weight gain was influenced by in-the-moment decisions rather than deliberate plans and action. Informed by experiences with weight before pregnancy and in previous pregnancies and information from health care providers, pregnant women form priorities which are not often congruent with active weight management. When faced with circumstantial barriers such as competing priorities, hunger, and pregnancy-related discomfort, weight management is not typically prioritized.

Given that pregnant women are typically highly motivated to make positive health-related behaviour changes, [45, 58, 59] we posit that the lack of prioritization of weight management reflects a lack of understanding that excess gestational weight gain has health implications. Only one of our participants understood this link, and that information was gleaned from a health care professional after trauma during a previous delivery. This finding is concordant with that from a recent systematic review reporting that few women understand the health risks of excess gestational weight gain, [45] with most women focused generally on “healthy eating” for the purpose of providing nutrients to the growing baby and many explicitly eschewing close weight monitoring [38, 45, 60–62].

Understanding the health risks of weight gain is important because it helps women make choices which promote weight management when those choices compete with other priorities. Participants in our study and others have enumerated the many other priorities which may make active weight management difficult: hunger, food preferences, childcare, limited financial resources, and limited time to prepare healthy food [35, 37, 39, 41–43, 60, 63, 64]. Many of these competing priorities are linked to the presence of other children, and our participants discussed this explicitly. This does not help explain our previous finding that nulliparity is predictive of higher weight gain, [46] although data on learning from weight gain in the first pregnancy may explain why multiparous women take weight gain more seriously.

Our previous study suggested that women who plan to gain more weight than the guidelines recommend are more likely to gain in excess. This is not a surprising finding, but the current study adds nuance to this finding. No participant reported aiming to knowingly gain more than the recommendation. Rather, they misinterpreted the guidelines, either mis-remembering the target weight or using a target weight for a different pre-pregnancy body type. This finding is congruent with other studies which have found that women who gained in excess of the guideline did not know how much they should gain, or cited a figure higher than the guidelines [35, 44, 64]. Our current study adds an explanatory factor to our previous predictive finding: Awareness of the guidelines is not enough; cursory conversations with health care providers may result in mis-application of the guidelines, or a lack of understanding about the health outcomes of excess or insufficient weight gain. In our qualitative sample, it was clear that health care providers were mentioning the guidelines, but not in a way which led women to understand the relationship between their weight-related practices and future health outcomes [36, 60, 65–71]. There is a previously documented discrepancy between pregnant women and provider perceptions of counselling about gestational weight gain, with most providers perceiving that they counsel about risks and benefits of weight gain and few pregnant women recalling counselling about that information [72, 73]. This suggests that there is room for more explicit information provision on this topic, perhaps written information which could be taken away and read at another time. Given the prevalence with which participants mis-remembered or mis-applied the guideline, a personalized handout showing what their own weight gain goal is, along with a trimester by trimester breakdown may be helpful.

Low feelings of self-efficacy over weight gain was predictive of gaining excess weight in our previous quantitative study [46]. Our current qualitative study offers that the perception of self-efficacy over weight gain a woman feels before pregnancy is likely to continue throughout the pregnancy. Most participants carried on their pre-pregnancy weight management practices, some loosened their previous practices and not a single participant became more active trying to control their weight. This sense of weight gain being uncontrollable is a common theme in the qualitative literature, especially among women who struggled with weight control prior to pregnancy [36–38, 44, 60, 63, 74]. Additional pregnancy-related physiological changes such as nausea, fatigue, cravings and physical discomfort may exacerbate perceptions of one’s body and appetite being out of control [35, 37, 62, 65, 67, 74–76]. Our qualitative findings showed that these competing priorities and embodied experiences played a significant role in influencing weight-related behaviour by regularly shifting in-the-moment decisions. Participants in our current study did not mention economic and structural circumstances (e.g. financial constraints, social pressures, safety concerns) which may further diminish perceptions of self-efficacy for many pregnant women [35, 37, 38, 40–44].

### Strengths and limitations

Strengths of this study include the emphasis on psychological and behavioral factors known to be predictive or protective of excess gestational weight gain, and the multi-disciplinary perspectives of the research team. Interviewing women at the end of pregnancy improved recall of recent behaviour and experiences, while still providing fulsome information about weight gain trajectory.

In this study, we relied on self-reported height and weight to calculate BMI measurements pre-pregnancy and during pregnancy. Women may not have provided accurate information, either because they did not know this information or chose not to provide it to the interviewer. We do not think the latter was prevalent, given the proportion of women who provided height and weight information that did not match their self-assessment of their weight category, e.g. describing self to be “normal” weight but providing measurements which correspond to an “overweight” BMI.

This study did not yield useful insight on all the predictive factors explored. All women reported regularly eating in front of a screen, most dismissed the notion that they were influenced by others who thought they should be eating for two, few discussed food as a way of controlling emotions, and we did not apply a formal measure of agreeableness.

Our sample had a higher proportion of midwifery clients (39%) than in the Ontario childbirthing population (18%) [77]. Our sample was more educated than Ontario women aged 25–44 years, with no participants who held a high school degree or less (18.3% of population), [78] and 28% who held a postgraduate or professional university degree (13.7% of population) [78]. Our sample was older than the typical population of Ontario women who give birth [79] and slightly more likely to identify as white (72% of participants vs 64% of Ontario women aged 25–44) [80]. However, in all of these respects *except* for proportion of midwifery clients, our sample resembled that of the earlier cohort study [46]. This suggests that pregnant women who volunteer to participate in research about weight gain may be more likely to be white, older, and more educated than the average pregnant Ontario woman. There may be transferability challenges when applying evidence about gestational weight gain in women who do not fit this profile.

## Conclusion

Despite the importance of gestational weight gain on key maternal and infant health indicators, few participants took a deliberate approach to planning and enacting healthy weight gain during pregnancy. Awareness of the Institute of Medicine gestational weight gain guidelines was not linked to correct understanding of how to apply the guideline, or a comprehension of the link between appropriate weight gain and health outcomes. This created a shaky foundation of knowledge that did not encourage in-the-moment choices which supported weight control when women encountered challenges related to competing priorities, hunger, and pregnancy discomfort. Despite regular weigh-ins during prenatal appointments, few women reported receiving notice from their care providers that their weight gain was a problem or receiving direction on how to manage weight gain. Lacking this information did not facilitate the adjustment of weight-related behaviours during pregnancy. Our findings suggest to health care providers that it is essential to counsel pregnant women about the health implications of weight gain in pregnancy, providing individualized information about how gestational weight gain guidelines apply to their pre-pregnancy BMI. Regular follow up conversations about weight gain over the course of pregnancy will provide essential information to influence in-the-moment decisions that impact weight gain.

## Supplementary information

**Additional file 1 Appendix 1**. Interview Guide

## Data Availability

The datasets generated and analyzed during the current study are not publicly available because participants did not consent to their data being shared beyond the research team.

## Abbreviations

BMI: Body mass index
FP: Family physician
GWG: Gestational weight gain
IOM: Institutes of medicine
lbs.: Pounds
MW: Midwife
OB: Obstetrician
HCP: Health care provider

## References

[1] Goldstein RF, Abell SK, Ranasinha S, Misso ML, Boyle JA, Harrison CL, Black MH, Li N, Hu G, Corrado F (2018). Gestational weight gain across continents and ethnicity: systematic review and meta-analysis of maternal and infant outcomes in more than one million women. BMC Med.

[2] Mannan M, Doi SA, Mamun AA (2013). Association between weight gain during pregnancy and postpartum weight retention and obesity: a bias-adjusted meta-analysis. Nutr Rev.

[3] Crane JM, White J, Murphy P, Burrage L, Hutchens D (2009). The effect of gestational weight gain by body mass index on maternal and neonatal outcomes. J Obstet Gynaecol Can.

[4] Nehring I, Schmoll S, Beyerlein A, Hauner H, von Kries R (2011). Gestational weight gain and long-term postpartum weight retention: a meta-analysis. Am J Clin Nutr.

[5] Siega-Riz AM, Viswanathan M, Moos MK, Deierlein A, Mumford S, Knaack J, Thieda P, Lux LJ, Lohr KN (2009). A systematic review of outcomes of maternal weight gain according to the institute of medicine recommendations: birthweight, fetal growth, and postpartum weight retention. Am J Obstet Gynecol.

[6] Cedergren M (2006). Effects of gestational weight gain and body mass index on obstetric outcome in Sweden. Int J Gynaecol Obstet.

[7] de la Torre L, Flick AA, Istwan N, Rhea D, Cordova Y, Dieguez C, Desch C, Gonzalez-Quintero VH (2011). The effect of new antepartum weight gain guidelines and prepregnancy body mass index on the development of pregnancy-related hypertension. Am J Perinatol.

[8] Mamun AA, Kinarivala M, O'Callaghan MJ, Williams GM, Najman JM, Callaway LK (2010). Associations of excess weight gain during pregnancy with long-term maternal overweight and obesity: evidence from 21 y postpartum follow-up. Am J Clin Nutr.

[9] Thorsdottir I, Gunnarsdottir I, Kvaran MA, Gretarsson SJ (2005). Maternal body mass index, duration of exclusive breastfeeding and children's developmental status at the age of 6 years. Eur J Clin Nutr.

[10] Hinkle SN, Sharma AJ, Swan DW, Schieve LA, Ramakrishnan U, Stein AD (2012). Excess gestational weight gain is associated with child adiposity among mothers with normal and overweight prepregnancy weight status. J Nutr.

[11] Poston L (2012). Maternal obesity, gestational weight gain and diet as determinants of offspring long term health. Best Pract Res Clin Endocrinol Metab.

[12] Sridhar SB, Darbinian J, Ehrlich SF, Markman MA, Gunderson EP, Ferrara A, Hedderson MM (2014). Maternal gestational weight gain and offspring risk for childhood overweight or obesity. Am J Obstet Gynecol.

[13] Flick AA, Brookfield KF, de la Torre L, Tudela CM, Duthely L, Gonzalez-Quintero VH (2010). Excessive weight gain among obese women and pregnancy outcomes. Am J Perinatol.

[14] Liu Y, Dai W, Dai X, Li Z (2012). Prepregnancy body mass index and gestational weight gain with the outcome of pregnancy: a 13-year study of 292,568 cases in China. Arch Gynecol Obstet.

[15] Herring SJ, Rose MZ, Skouteris H, Oken E (2012). Optimizing weight gain in pregnancy to prevent obesity in women and children. Diabetes Obes Metab.

[16] Lucia Bergmann R, Bergmann KE, Haschke-Becher E, Richter R, Dudenhausen JW, Barclay D, Haschke F (2007). Does maternal docosahexaenoic acid supplementation during pregnancy and lactation lower BMI in late infancy?. J Perinat Med.

[17] Nohr EA, Villamor E, Vaeth M, Olsen J, Cnattingius S (2012). Mortality in infants of obese mothers: is risk modified by mode of delivery?. Acta Obstet Gynecol Scand.

[18] Nesbitt TS, Gilbert WM, Herrchen B (1998). Shoulder dystocia and associated risk factors with macrosomic infants born in California. Am J Obstet Gynecol.

[19] Chung JG, Taylor RS, Thompson JM, Anderson NH, Dekker GA, Kenny LC, McCowan LM, Consortium S (2013). Gestational weight gain and adverse pregnancy outcomes in a nulliparous cohort. Eur J Obstet Gynecol Reprod Biol.

[20] McDonald SD, Woolcott C, Chapinal N, Guo Y, Murphy P, Dzakpasu S (2018). Interprovincial variation in pre-pregnancy body mass index and gestational weight gain and their impact on neonatal birth weight with respect to small and large for gestational age. Can J Public Health.

[21] Durie DE, Thornburg LL, Glantz JC (2011). Effect of second-trimester and third-trimester rate of gestational weight gain on maternal and neonatal outcomes. Obstet Gynecol.

[22] Kowal C, Kuk J, Tamim H (2012). Characteristics of weight gain in pregnancy among Canadian women. Matern Child Health J.

[23] Simas TA, Liao X, Garrison A, Sullivan GM, Howard AE, Hardy JR (2011). Impact of updated Institute of Medicine guidelines on prepregnancy body mass index categorization, gestational weight gain recommendations, and needed counseling. J Women's Health.

[24] Fell DB, Joseph KS, Dodds L, Allen AC, Jangaard K, Van den HM (2005). Changes in maternal characteristics in Nova Scotia, Canada from 1988 to 2001. Can J Public Health.

[25] Deputy NP, Sharma AJ, Kim SY, Hinkle SN (2015). Prevalence and characteristics associated with gestational weight gain adequacy. Obstet Gynecol.

[26] Mujahid MS, Roux AV, Borrell LN, Nieto FJ (2005). Cross-sectional and longitudinal associations of BMI with socioeconomic characteristics. Obes Res.

[27] Manios Y, Panagiotakos DB, Pitsavos C, Polychronopoulos E, Stefanadis C (2005). Implication of socio-economic status on the prevalence of overweight and obesity in Greek adults: the ATTICA study. Health Policy.

[28] Sundquist J, Winkleby M (2000). Country of birth, acculturation status and abdominal obesity in a national sample of Mexican–American women and men. Int J Epidemiol.

[29] Ellaway A, Anderson A, Macintyre S (1997). Does area of residence affect body size and shape?. Int J Obes.

[30] Cubbin C, Hadden WC, Winkleby MA (2000). Neighborhood context and cardiovascular disease risk factors: the contribution of material deprivation. Ethn Dis.

[31] Kress AM, Peterson MR, Hartzell MC (2006). Association between obesity and depressive symptoms among US military active duty service personnel, 2002. J Psychosom Res.

[32] Jorm AF, Korten AE, Christensen H, Jacomb PA, Rodgers B, Parslow RA (2003). Association of obesity with anxiety, depression and emotional well-being: a community survey. Aust N Z J Public Health.

[33] Räikkönen K, Matthews K, Kuller L. Anthropometric and psychosocial determinants of visceral obesity in healthy postmenopausal women. Int J Obes Relat Metab Disord. 1999;23(8):775–82. 10.1038/sj.ijo.0800917 10490776

[34] Lallukka T, Laaksonen M, Martikainen P, Sarlio-Lähteenkorva S, Lahelma E (2005). Psychosocial working conditions and weight gain among employees. Int J Obes.

[35] Black TL, Raine K, Willows ND (2008). Understanding prenatal weight gain in first nations women. Can J Diabetes.

[36] Garnweidner LM, Pettersen KS, Mosdol A (2013). Experiences with nutrition-related information during antenatal care of pregnant women of different ethnic backgrounds residing in the area of Oslo, Norway. Midwifery.

[37] Groth SW, Morrison-Beedy D (2013). Low-income, pregnant, African American women's views on physical activity and diet. J Midwifery Womens Health.

[38] Groth SW, Morrison-Beedy D, Meng Y (2012). How pregnant African American women view pregnancy weight gain. J Obstet Gynecol Neonatal Nurs.

[39] Jette S, Rail G (2014). Resisting, reproducing, resigned? Low-income pregnant women's discursive constructions and experiences of health and weight gain. Nurs Inq.

[40] Krans EE, Chang JC (2011). A will without a way: barriers and facilitators to exercise during pregnancy of low-income, African American women. Women Health.

[41] Reyes NR, Klotz AA, Herring SJ (2013). A qualitative study of motivators and barriers to healthy eating in pregnancy for low-income, overweight, African-American mothers. J Acad Nutri Diet.

[42] Thomas M, Vieten C, Adler N, Ammondson I, Coleman-Phox K, Epel E, Laraia B (2014). Potential for a stress reduction intervention to promote healthy gestational weight gain: focus groups with low-income pregnant women. Womens Health Issues.

[43] Thornton PL, Kieffer EC, Salabarria-Pena Y, Odoms-Young A, Willis SK, Kim H, Salinas MA (2006). Weight, diet, and physical activity-related beliefs and practices among pregnant and postpartum Latino women: the role of social support. Matern Child Health J.

[44] Vallianatos H, Brennand EA, Raine K, Stephen Q, Petawabano B, Dannenbaum D, Willows ND (2006). Beliefs and practices of first nation women about weight gain during pregnancy and lactation: implications for women's health. Can J Nurs Res.

[45] Vanstone M, Kandasamy S, Giacomini M, DeJean D, McDonald SD (2017). Pregnant women's perceptions of gestational weight gain: a systematic review and meta-synthesis of qualitative research. Matern Child Nutr.

[46] McDonald SD, Yu M, Van Blyderveen S, Schmidt L, Sword W, Vanstone M, Biringer A, Beyene J. Prediction of excess pregnancy weight gain using psychological, physical, and social predictors: a validated model in a prospective cohort study. PLoS One. 2020;15(6):e0233774. 10.1371/journal.pone.0233774. 10.1371/journal.pone.0233774 PMC7266315 32484813

[47] Creswell JW, Clark VLP. Designing and conducting mixed methods research. Thousand Oaks: Sage publications; 2017.

[48] Sandelowski M (2000). Whatever happened to qualitative description?. Res Nurs Health.

[49] Sandelowski M (2010). What's in a name? Qualitative description revisited. Res Nurs Health.

[50] Neergaard MA, Olesen F, Andersen RS, Sondergaard J (2009). Qualitative description–the poor cousin of health research?. BMC Med Res Methodol.

[51] Crotty M. The foundations of social research: meaning and perspective in the research process. Thousand Oaks: Sage; 1998.

[52] Saunders B, Sim J, Kingstone T, Baker S, Waterfield J, Bartlam B, Burroughs H, Jinks C (2018). Saturation in qualitative research: exploring its conceptualization and operationalization. Qual Quant.

[53] Sandelowski M, Givens LM (2008). Theoretical saturation. The Sage encyclopedia of qualitative methods.

[54] Institute of Medicine and National Research Council of the National Academies (2009). Weight gain during pregnancy: reexamining the guidelines.

[55] Elo S, Kyngäs H (2008). The qualitative content analysis process. J Adv Nurs.

[56] Charmaz K. Constructing grounded theory. Thousand Oaks: Sage; 2014.

[57] Flick U, Flick U, von Kardoff E, Steinke I (2004). Triangulation in qualitative research. A companion to qualitative research.

[58] Atkinson L, Shaw RL, French DP (2016). Is pregnancy a teachable moment for diet and physical activity behaviour change? An interpretative phenomenological analysis of the experiences of women during their first pregnancy. Br J Health Psychol.

[59] McBride CM, Emmons KM, Lipkus IM (2003). Understanding the potential of teachable moments: the case of smoking cessation. Health Educ Res.

[60] Heery E, McConnon A, Kelleher CC, Wall PG, McAuliffe FM (2013). Perspectives on weight gain and lifestyle practices during pregnancy among women with a history of macrosomia: a qualitative study in the Republic of Ireland. BMC Pregnancy Childbirth.

[61] Keely A, Gunning M, Denison F (2011). Maternal obesity in pregnancy: Women's understanding of risks. Br J Midwifery.

[62] Olander EK, Atkinson L, Edmunds JK, French DP (2011). The views of pre- and post-natal women and health professionals regarding gestational weight gain: an exploratory study. Sex Reprod Healthc.

[63] Paul KH, Graham ML, Olson CM (2013). The web of risk factors for excessive gestational weight gain in low income women. Matern Child Health J.

[64] Tovar A, Chasan-Taber L, Bermudez OI, Hyatt RR, Must A (2010). Knowledge, attitudes, and beliefs regarding weight gain during pregnancy among Hispanic women. Matern Child Health J.

[65] Arden MA, Duxbury AMS, Soltani H (2014). Responses to gestational weight management guidance: a thematic analysis of comments made by women in online parenting forums. Bmc Pregnancy Childbirth.

[66] Duthie EA, Drew EM, Flynn KE (2013). Patient-provider communication about gestational weight gain among nulliparous women: a qualitative study of the views of obstetricians and first-time pregnant women. BMC Pregnancy Childbirth.

[67] Furness PJ, McSeveny K, Arden MA, Garland C, Dearden AM, Soltani H. Maternal obesity support services: a qualitative study of the perspectives of women and midwives. Bmc Pregnancy Childbirth. 2011;11. 10.1186/1471-2393-11-69 PMC3198957 21982306

[68] Herring SJ, Henry TQ, Klotz AA, Foster GD, Whitaker RC (2012). Perceptions of low-income African-American mothers about excessive gestational weight gain. Matern Child Health J.

[69] Lindhardt CL, Rubak S, Mogensen O, Lamont RF, Joergensen JS (2013). The experience of pregnant women with a body mass index >30 kg/m2 of their encounters with healthcare professionals. Acta Obstet Gynecol Scand.

[70] Mills A, Schmied VA, Dahlen HG: 'Get alongside us', women's experiences of being overweight and pregnant in Sydney, Australia**.** Matern Child Nutr2013, 9(3):309–321. 10.1111/j.1740-8709.2011.00386.x PMC6860714 22168548

[71] Stengel MR, Kraschnewski JL, Hwang SW, Kjerulff KH, Chuang CH (2012). "What my doctor didn't tell me": examining health care provider advice to overweight and obese pregnant women on gestational weight gain and physical activity. Womens Health Issues.

[72] Lutsiv O, Bracken K, Pullenayegum E, Sword W, Taylor VH, McDonald SD (2012). Little congruence between health care provider and patient perceptions of counselling on gestational weight gain. J Obstet Gynaecol Can.

[73] McDonald SD, Pullenayegum E, Taylor VH, Lutsiv O, Bracken K, Good C, Hutton E, Sword W (2011). Despite 2009 guidelines, few women report being counseled correctly about weight gain during pregnancy. Am J Obstet Gynecol.

[74] Harper EA, Rail G (2012). 'Gaining the right amount for my baby': Young pregnant women's discursive constructions of health. Health Sociol Rev.

[75] Goodrich K, Cregger M, Wilcox S, Liu J (2013). A qualitative study of factors affecting pregnancy weight gain in African American women. Matern Child Health J.

[76] Wennberg AL, Lundqvist A, Hogberg U, Sandstrom H, Hamberg K (2013). Women's experiences of dietary advice and dietary changes during pregnancy. Midwifery.

[77] Association of Ontario Midwives. Midwifery by the numbers. [Internet]. Toronto: Association of Ontario Midwives. [cited 2020 August 29]. Available from: https://www.ontariomidwives.ca/midwifery-numbers.

[78] Statistics Canada: Education Highlight Tables, 2016 Census. In: Census of the Population, 2016. vol. 98–304-X, 2019-02-20 edn. Ottawa, ON; 2016.

[79] Statistics Canada. Fertility: overview 2012–2016. In: Report on the Demographic Situation in Canada. vol 91-209-X. 2018-06-05. Ottawa: Government of Canada; 2018.

[80] Statistics Canada: Visible Minority, 2016 Census. In: Census of the Population, 2016. vol. 98–304-X, 2019-02-20 edn. Ottawa, ON; 2016.

