# Supplementary material for: Competing priorities: a qualitative study of how women make and enact decisions about weight gain in pregnancy
Source: BMC Pregnancy Childbirth. 2020 Sep 3;20:507. doi: 10.1186/s12884-020-03210-5 (PMC7470685; doi:10.1186/s12884-020-03210-5)
Supplement: Supplementary file 1 — Additional file 1 Appendix 1. Interview Guide [file 12884_2020_3210_MOESM1_ESM.docx]

Appendix 1: Interview Guide

Intro: Introduce myself, the study, consent form, etc.

1. **To begin, tell me a bit about your pregnancy. How have things been going for you?** *Note: This question is intended to help establish rapport*

*So as you know our study is about weight gain in pregnancy. I just want to reiterate that there won’t be any judgement here and I hope that you are comfortable sharing your experiences with me. Your insights will help us to better understand women’s ideas about weight gain and the different factors that influence the amount of weight gained. If you’re ready, we’re going to jump into the weight related questions, okay?*

1. ***Is this your first pregnancy? [nulliparity]***

*Follow up for multiparous women: Was weight something you were thinking about in your first pregnancy? Was it something you discussed with your doctor, nurse or midwife?*

*After pregnancy, how did weight loss go?*

*Anything you plan to do differently about weight gain in this pregnancy?*

1. **Before becoming pregnant, did you talk to anyone about how to have a healthy pregnancy? [planned GWG]**

*Prompt: food, physical activity*

*Follow up: Who did you talk to?*

1. **Did you have any questions about weight gain early on? [planned GWG, GWG knowledge, GWG knowledge sources]**

*Probe: Who/what/where did you go to for answers?*

*What advice did you get about food, nutrition, and physical activity?*

1. **Early on in this pregnancy, did you have a weight gain goal? [planned GWG]**

*Probe: How did you come up with it/them?*

*Has it changed since then? If it has changed, what factors led you to change your goal?*

*What kinds of things are you doing to achieve that goal?*

1. **Do you feel like you are in control of your weight gain? [perceived control of GWG—really self –efficacy ]**

*Probe: If not, what elements are outside of your control?*

*Note to self: ensure participants verbalize an answer. If participants say “well I guess I’m kind of in control”, push further- what elements are they in control of, what elements are outside of their control?*

1. **Tell me about how meal planning and preparation happens for you. [Perceived control of GWG]**
   1. *Do you shop and cook yourself? Is there someone else who does this? [if someone else has a main role- probe that further. Do they make choices you are happy with? Do you influence these choices?]*
   2. *How often do you eat at a restaurant or eat take out?*
2. **Do you track your weight? [control of GWG, planned GWG]**

*Probe: Does your doctor, nurse, or midwife track your weight?*

*Do you talk about it together?*

1. **What do you do when you are hungry? [control of GWG]**

*Follow up: What if it’s not a meal time? What if it’s an inconvenient time to eat, e.g. in the middle of the work day?*

*Do you keep food on hand or wait until a meal time?*

1. **When you are upset, what do you usually do to make yourself feel better? [emotional control]**

*Probe: What do you do?*

*What if that doesn’t help? What do you do then?*

*[If not mentioned] Do you ever use food to feel better? Has this changed since becoming pregnant?*

*Follow up: Is that different from before you became pregnant?*

1. **What does “eating for two” mean to you? [eating for 2]**

*Probe: Do you hear it often? From who? When? Why? Prompt for story*

1. **What do you think it means to the people who are saying it? [eating for 2]**
2. **If someone were to serve you something you know might not be the healthiest choice for you, how would you handle that? [agreeableness]**
3. **Did you try and control your weight before pregnancy? [conscientiousness]**
   1. *In what ways?*
   2. *Did you like the way your body looked before pregnancy?*

*Follow up: Has any of that changes since becoming pregnant?*

1. **Sometimes we tend to compensate for certain behaviours, for example, if I eat a few extra cookies, I might take the stairs instead of the elevator. Have you used any kinds of compensation to justify weight gain during pregnancy? [disordered eating]**

*Prompt: Do you ever feel guilty about what you have eaten, or about the physical activity that you haven’t done? If so, what do you do when you have those feelings of guilt?*

*What else do you tell yourself in those moments?*

1. **Sometimes people who are unhappy with the way their body looks use more significant ways to try to change this. Some women have told us about using laxatives to lose weight, or drastically restricting the types or quantity of food they eat. Is this something you have experienced, either before or during pregnancy?**
2. **Do you ever eat in front of a screen?**

*Probe: breakfast, lunch, dinner. Who else eats with you? What if you’re eating alone?*

**Demographics: Pre-pregnancy height and weight, Current weight, Due date, Age, Highest level of education, Occupation, Race, Healthcare provider, Previous births**

That’s the end of our interview. I want to thank you for your time, and thank you for sharing your experiences with me. I know it takes a level of trust to do that and I’m so grateful that you’ve entrusted me with your thoughts, feelings, and experiences. I hope you have a wonderful rest of your day, and if you don’t have any more questions, you are welcome to leave whenever you’re ready.
